# Calibration of a Constitutive Model from Tension and Nanoindentation for Lead-Free Solder

**DOI:** 10.3390/mi9110608

**Published:** 2018-11-20

**Authors:** Xu Long, Xiaodi Zhang, Wenbin Tang, Shaobin Wang, Yihui Feng, Chao Chang

**Affiliations:** 1 School of Mechanics and Civil & Architecture, Northwestern Polytechnical University, Xi’an 710072, China; 2 College of Mining Engineering, Liaoning Shihua University, Fushun 113001, China; zhangxiaodiw@163.com; 3 School of Mechanics and Civil & Architecture, Northwestern Polytechnical University, Xi’an 710072, China; tangwb@mail.nwpu.edu.cn; 4 School of Mechanics and Civil & Architecture, Northwestern Polytechnical University, Xi’an 710072, China; shaobinwang@mail.nwpu.edu.cn; 5 State Key Laboratory of Nonlinear Mechanics, Institute of Mechanics, Chinese Academy of Sciences, Beijing 100190, China; fengyh@lnm.imech.ac.cn; 6 School of Applied Science, Taiyuan University of Science and Technology, Taiyuan 030024, China; cc@tyust.edu.cn

**Keywords:** nanoindentation, constitutive model, rate factor, dimensionless analysis, solder

## Abstract

It is challenging to evaluate constitutive behaviour by using conventional uniaxial tests for materials with limited sizes, considering the miniaturization trend of integrated circuits in electronic devices. An instrumented nanoindentation approach is appealing to obtain local properties as the function of penetration depth. In this paper, both conventional tensile and nanoindentation experiments are performed on samples of a lead-free Sn–3.0Ag–0.5Cu (SAC305) solder alloy. In order to align the material behaviour, thermal treatments were performed at different temperatures and durations for all specimens, for both tensile experiments and nanoindentation experiments. Based on the self-similarity of the used Berkovich indenter, a power-law model is adopted to describe the stress–strain relationship by means of analytical dimensionless analysis on the applied load-penetration depth responses from nanoindentation experiments. In light of the significant difference of applied strain rates in the tensile and nanoindentation experiments, two “rate factors” are proposed by multiplying the representative stress and stress exponent in the adopted analytical model, and the corresponding values are determined for the best predictions of nanoindentation responses in the form of an applied load–indentation depth relationship. Eventually, good agreement is achieved when comparing the stress–strain responses measured from tensile experiments and estimated from the applied load–indentation depth responses of nanoindentation experiments. The rate factors $\psi_{\sigma }$ and $\psi_{n}$ are calibrated to be about 0.52 and 0.10, respectively, which facilitate the conversion of constitutive behaviour from nanoindentation experiments for material sample with a limited size.

## 1. Introduction

As described by the observation of Moore’s law, the miniaturization of electronic devices is still continuously ongoing, despite material and manufacturing challenges [1]. It is difficult to obtain the constitutive behaviour of new emerging electronic packaging materials at such a local scale. Even though conventional tensile experiments can be performed to obtain the stress–strain relationship, the specimens have to be designed with a sufficient size to be conveniently clamped. The requirement of specimen geometry is difficult to meet for some die-attach pastes, such as silver nanoparticle paste, with the evaporation during the sintering process. The microstructure and also the material properties of sintered material in a great volume is distinguishingly different from the die-attach form in actual applications, due to the coalescence of sintered nanoparticles [2]. The material properties of lead-containing solders have been well-known and applied with an outstanding mechanical reliability; nevertheless, these solders have been replaced by lead-free solder alloys in recent years around the world. Compared with lead-containing solders, the lead-free alloys’ capacity to resist thermo-mechanical fatigue and electromigration is better, but is also detrimental to mechanical shock and whisker growth [3]. As reviewed by Zhang and Tu [4], extensive studies on the composite Pb-free solders by adding nanoparticles has been conducted to strengthen the physical and solder mechanical properties, such as wettability, creep resistance, and hardness. Xu et al. [5] demonstrated that the Sn–Ag–Cu solder paste added by FeCo magnetic nanoparticles can be reflowed locally with alternating current magnetic fields, so that the interconnects form in area array packages, and the eddy current heating in the printed circuit board is minimized.

In light of the miniaturization trend of electronic devices, it is challenging to obtain the constitutive behaviour by using conventional uniaxial tests for small-sized material samples, as adopted during packaging applications. Instrumented nanoindentation is a suitable approach to quantify the material properties at a smaller scale, which is feasible for the die-attach materials, especially with a limited size of electronic devices. Long et al. [6] determined the strain-rate sensitivity of several types of die-attached materials, using nanoindentation with multiple strain-rate jumps accompanied by a continuous stiffness measurement. Zhang et al. [7] investigated the size effect of surface pit defects on the yield load of thin film, by using the quasi-continuum method to simulate nanoindentation. Bo et al. [8] determined the mechanical properties of cells using the stress–relaxation curve from the indentation process with an atomic force microscope. Recently, Rengel et al. [9] measured the mechanical behaviour of a hydride blister to reduce the mechanical and fracture properties of nuclear fuel cladding. Lee et al. [10] performed nanoindentation under the temperature range between 25 °C and 300 °C to determine the activation energy for the plastic flow in a nanocrystalline CoCrFeMnNi high-entropy alloy. Chu, et al. [11] investigated the mechanical properties of Fe–Zr welded joints, as well as dependence with the microstructures using nanoindentation. Hsueh et al. [12] revealed the size effect and strain-induced double twin in duplex stainless steel, prepared using the activated gas tungsten arc welding technique. Using both indentation and tensile tests for fully dense nanocrystalline nickel, Schwaiger et al. [13] found that the strain-rate sensitivity of deformation is strongly related to the grain size. Phani and Oliver [14] demonstrated that uniaxial creep behaviour over a wide range of strain rates and temperatures agrees well with the uniaxial creep behavior using high temperature nanoindentation. Humphrey and Jankowski [15] measured and compared the strain-rate dependence of the tensile strength on the grain size in crystalline nickel foils, using both tensile and micro-scratch methods. However, it is controversial to correlate the constitutive behaviour measured by tensile tests with the material behaviour measured by nanoindentations, despite the intensive investigations of mechanical properties measured using the instrumented nanoindentation approach [16,17,18,19,20]. In terms of the equivalence of measured material constitutive relationships, it is rare to further quantitatively investigate the constitutive behaviour obtained from tension and indentation methods. 

This discrepancy in stress–strain relationships obtained from tension and indentation experiments motivated this paper, in order to find a reliable approach for consistently estimating the constitutive properties from nanoindentation experiments, by calibrating the involved material parameters against the results from tensile experiments. As the aim of this study, the constitutive behaviour measured by nanoindentations is convincingly used for finite element simulations, to examine the mechanical reliability of electronic packaging structures rather than performing tension experiments with the time-consuming preparation of tension samples, which are probably of a size that does not comply with the actual applications. By emphasizing the electronic packaging applications, a representative, lead-free Sn–3.0Ag–0.5Cu (in wt. %; SAC305) solder material is employed to provide a uniform matrix under nanoindentation, in order to rule out the microstructure effect. The solder alloy SAC305 is a typical strain-rate-sensitive, visco-plastic material, and deemed as one of the potential alternatives for consumer electronics [21,22]. Nevertheless, it should be pointed out that the proposed approach herein is generalized for metals and alloys, provided that the prepared material samples have a dense microstructure without significant residual stress.

## 2. Sample Preparation and Experimental Setup

**Figure 1:**
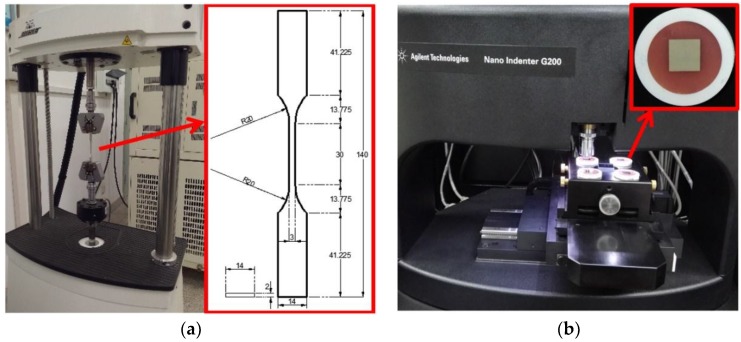
Experimental equipment and material sample. (**a**) Tensile experiment (unit: mm); (**b**) nanoindentation experiment.

The SAC305 bulk solder alloy is manufactured by Alpha Assembly Solutions (South Plainfield, NJ, USA) to be free of cast in impurities or included oxides. The SAC305 solder samples for tensile and nanoindentation experiments were prepared in the form of a dog bone and mounted plate, as shown in Figure 1a,b, respectively. The experimental equipment were a Bose ElectroForce 3330 mechanical test machine and a Nano Indenter G200 by Agilent Technologies (Santa Clara, CA, USA). The bulk solder was machined to achieve the desired dog-bone type of specimen shown in Figure 1a, which was designed by referring to American Society for Testing and Materials (ASTM) E8/E8M [23]. The samples for nanoindentations were approximately 10.0 mm × 10.0 mm × 2.0 mm, and were mounted in polyvinyl chloride (PVC) tubes by dental base acrylic resin powder, as shown in the magnified inset of Figure 1b. Despite being from the same material source, thermal treatment was applied in order to align the material property for both types of specimens, by using an air furnace with a temperature stability of ±1.0 °C. This thermal treatment is important for avoiding the effect of micro-defects and residual stress on the mechanical properties of the material sample of interest when correlating the experimental results from tensile and nanoindentation experiments. As found by the authors [24,25], the thermal treatment minimizes the residual stress and stabilizes the mechanical property of the annealed solder. In this study, the applied annealing temperatures were 80 °C, 125 °C, 165 °C, and 210 °C, and the durations were 2 h, 6 h, 12 h, 24 h, and 48 h. It should be noted that the high-temperature annealing temperature at 210 °C is slightly lower than the melting point *T*_m_ of 217 °C for SAC305 solder.

The applied strain rate of tensile experiments is 5 × 10^−4^ s^−1^ under displacement control, in order to obtain the quasi-static behavior of SAC305 solder material. The stress–strain relationships can be directly obtained and readily adopted for finite element simulations of electronic packaging structures. In nanoindentation experiments, using a diamond Berkovich indenter (Agilent Technologies, Santa Clara, CA, USA) in the shape of a three-sided pyramid, the strain rate of 0.05 s^−1^ is applied, with the maximum indentation depth of 2000 nm, to eliminate the influence of surface roughness. It should be noted that if a much lower strain rate is applied, to be compatible with the value of tensile experiments, an indentation will take a few hours or even more. This is extremely time-consuming, and also significantly deteriorates the accuracy of nanoindentation results (such as the determination of the contact area), due to the limitation of thermal drift correction of the nanoindentation instrument. 

**Figure 2:**
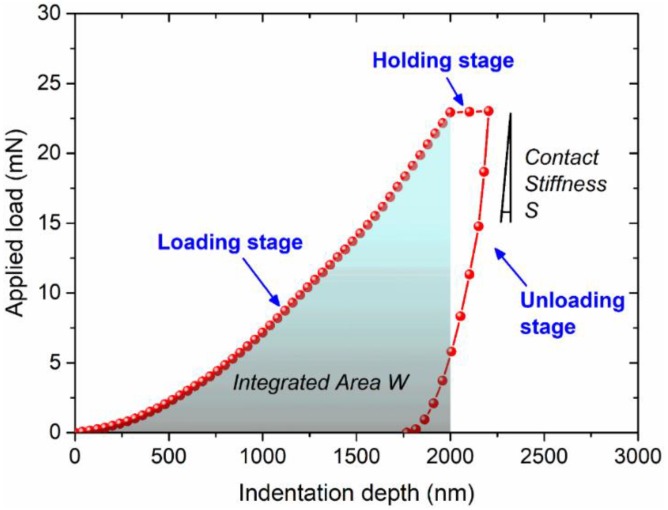
Typical nanoindentation response in the form of applied load–indentation depth.

For each indentation, the applied load–indentation depth response can be divided into three stages, as shown in Figure 2—that is, the loading, holding, and unloading stages. By controlling the indenter speed at various penetration depths, the strain rate of 0.05 s^−1^ is maintained at the temperature of 27 °C, until the maximum depth of 2000 nm. Later, the obtained load–depth curves for repeated experiments are averaged to objectively measure the mechanical properties of SAC305 material. Figure 2 shows that the highlighted area *W* of loading stage can be calculated by integrating the response during the penetration between 0 nm and 2000 nm, and also the contact stiffness *S* can be determined from the initial slope of the applied load–indentation depth response. 

## 3. Experimental Results

### 3.1. Averaged Nanoindentation Response

**Figure 3:**
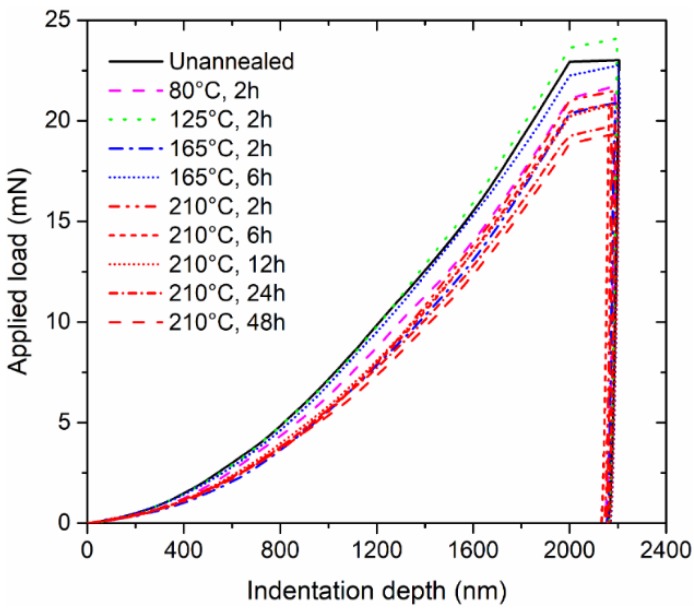
Averaged applied load–indentation depth response to nanoindentation.

At least five indentations were performed for each sample among the various annealing treatments. The averaged nanoindentation responses, in the form of applied load–indentation depth curves, are summarized in Figure 3. It was found that annealing treatment is capable of affecting the mechanical behaviour of material under indentation. According to the findings for other metal and alloy materials [26,27], residual stress can be eliminated to a certain extent after the thermal treatment at a high temperature, which will stabilize the microstructure and also the subsequent mechanical properties. Consistent with findings in the literature, the annealing temperature of 210 °C in this study led to a consistent reduction of nanoindentation response (as shown in Figure 3) compared with the unannealed and other annealing temperature conditions, because the applied temperature of 210 °C is closer to the melting point (i.e., 217 °C for SAC305 solder). Additionally, it was also observed that a longer thermal treatment is more effective. Therefore, it is believed that the annealing effect results from thermal accumulation in the form of an equivalent mass diffusion, if the given temperature is sufficiently high. The input energy promotes the alleviation of micro-scale defects to achieve a homogenous eutectic microstructure. Meanwhile, the induced increase of grain size—and thus, a coarser microstructure—will decrease the resistance to dislocation motion, lower yield strength, and working hardening rate [28,29].

### 3.2. Young’s Modulus and Hardness

Based on the continuous stiffness measurement [30], Young’s modulus and hardness can be measured as functions of indentation depths, by superimposing a small oscillation to the indentation load controlled by a frequency-specific amplifier to ensure constant amplitude and driving frequency. Based on the harmonic oscillator, the stiffness $K_{c}$ is provided in Equation (1) by Hay et al. [30]:

(1) $$\;K_{c}=1/\left[\frac{1}{\left(F_{0}/z_{0}\right)\cos\phi -{\left(F_{0}/z_{0}\right)\cos\phi |}_{free}}-\frac{1}{K_{f}}\right]\;$$

where $K_{f}$ is the elastic stiffness of the frame; ϕ is the phase angle by which the response lags the excitation; and $z_{0}/F_{0}$ is the dynamic compliance, to represent the ratio of the displacement oscillation to the applied excitation. The subscript *free* indicates that the natural frequency of the indenter is in its free-hanging state, so the term ${\left(F_{0}/z_{0}\right)\cos\phi |}_{free}$ can be determined as $K-m\omega^{2}$, where K is the stiffness of the spring supporting the indenter shaft, m is the indenter mass, and ω=2πf represents the angular frequency of the indenter oscillates. Therefore, the reduced Young’s modulus $E_{r}$ and hardness H can be determined by Equations (2) and (3) [31]:

(2) $$\;E_{r}=\frac{\sqrt{\pi }}{2\beta }\frac{K_{c}}{\sqrt{A_{c}}}\;$$

(3) $$\;H=\frac{P}{A_{c}}\;$$

where $A_{c}=24.56h_{c}^{2}$, and is the projected contact area at the contact depth $h_{c}$; the shape constant is β=1.034 for a Berkovich indenter; and P is the indentation load. The Young’s modulus E can be further calculated by Equation (4).

(4) $$\;E=\left(1-\nu^{2}\right)/\left[\frac{1}{E_{r}}-\frac{1-\nu_{d}^{2}}{E_{d}}\right]\;$$

where ν is 0.42 for the Poisson’s ratio of SAC305, and $\nu_{d}$ and $E_{d}$ are the Poisson’s ratio and Young’s modulus of diamond, respectively, for the used Berkovich indenter, and are taken as 0.07 and 1140 GPa. 

**Figure 4:**
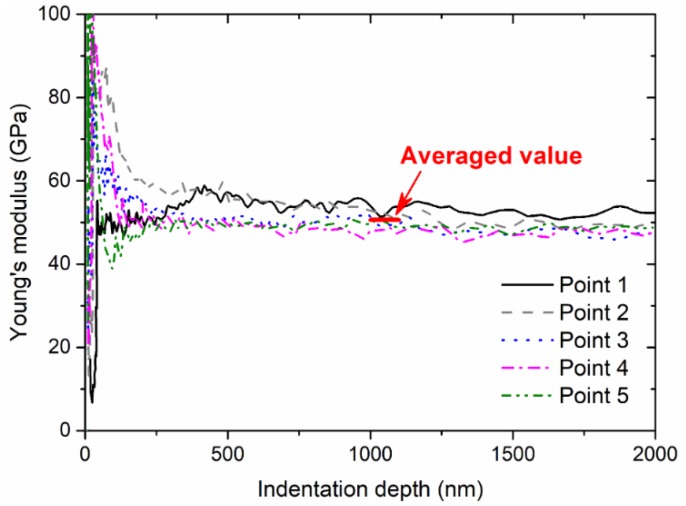
Measured value of Young’s modulus as a function of indentation depth.

**Figure 5:**
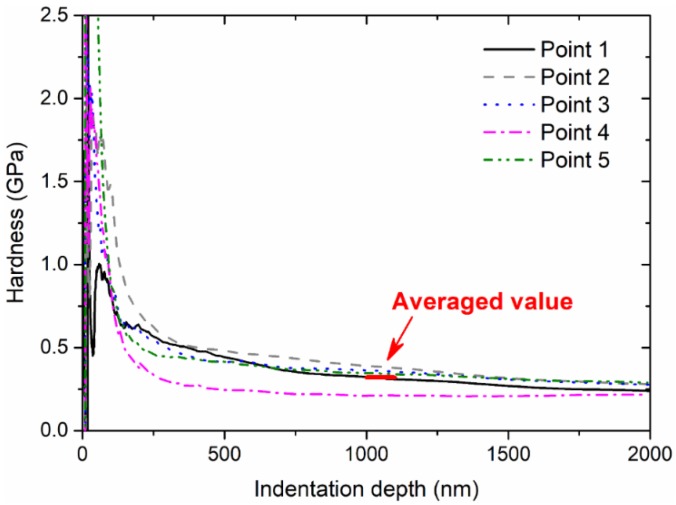
Measured value of hardness as a function of indentation depth.

**Figure 6:**
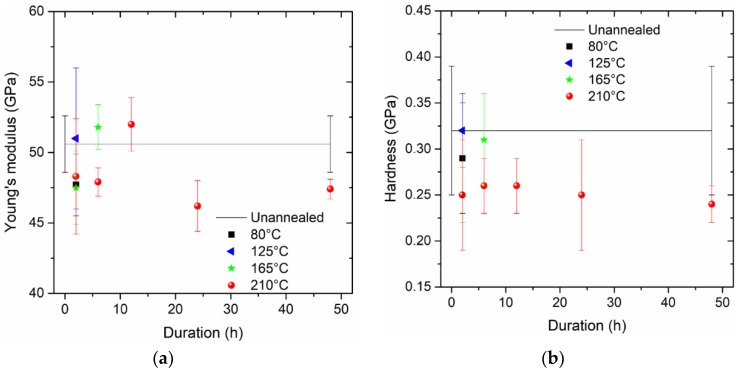
Mechanical properties of the Sn–3.0Ag–0.5Cu (SAC305) solder: (**a**) Young’s modulus; (**b**) hardness.

As shown in Figure 4 and Figure 5, these values of Young’s modulus and hardness are stabilized after the initial indentation depth of 1000 nm, with some effects due to subtraction, surface stress, and roughness. In the present study, the Young’s modulus and hardness can obtained by averaging the corresponding values between 1000 nm and 1100 nm. As summarized in Figure 6, the Young’s modulus seems to be more random compared with hardness. The thermal treatment at a higher temperature intends to decrease hardness significantly and consistently, while the treatment duration does not dominate this effect. However, it should be noted that pile-up deformation of indentations may induce the greater elastic modulus calculated from indentation responses, despite some alleviation approaches to recover the linearity of the indentation load and the square of the indentation depth; these alleviation approaches may be done by removing the initial part of the measured load–indentation depth curves, especially for shallow indentation depths. 

## 4. Theoretical Analysis

By focusing on the essential relationships between different physical quantities, dimensional analysis has been widely applied in engineering and science areas to identify physical meanings and measurement units, and track these dimensions during formula derivations. In order to reveal the intrinsic mechanism, the dimensionless approach proposed by Ogasawara et al. [32] was adopted herein, due to its fewer parameters with clear physical meanings. The constitutive model in form of a power law $\sigma_{R}\langle \varepsilon_{R}\rangle =R{\left(\varepsilon^{e}+\varepsilon_{R}\right)}^{n}$ is parameterized based on dimensionless analysis, in which $\sigma_{R}$ is the representative stress; R and n are the hardening rates and exponent, respectively; $\varepsilon^{e}=\sigma /E$ is the elastic strain; and $\varepsilon_{R}$ is the representative strain, defined as the plastic strain during axisymmetric deformation. The essence of the proposed approach is provided in Equations (5) and (6) for characterizing the dominate information in the loading and unloading parts of the applied load–penetration depth curve, respectively. However, enrichments are made for the parameters $\sigma_{R}$ and n that take into account the strain rate effect, as discussed below.

Tensile experiments emphasize the macroscopic-scale deformation behaviour of materials as the average over a great number of microstructural length scales and features, while nanoindentation experiments focus on the local-scale characteristics. In fact, a good agreement can be made between nanoindentation and uniaxial experiments by controlling the indentation strain, using the ratio of loading rate and the applied load proposed by Lucas and Oliver [33]. Atkins and Tabor [34] introduced the concept of representative indentation strain to compare the indentation experiments with uniaxial experiments. They found that the constraint factor is greatly dependent on strain rate, and may lead to a significant discrepancy between the indentation and uniaxial experiments. As compared by Maier et al. [35], the strain rate sensitivity measured by indentation tests is in good agreement with that measured by uniaxial compression tests. Obviously, the strain rates in the tensile and nanoindentation experiments in this study are different, so the strain rate effect is evaluated with further enrichment to unify the constitutive behavior, as shown in Equations (5) and (6) by multiplying the rate factors $\psi_{\sigma }$ and $\psi_{n}$ with the parameters $\sigma_{R}$ and n, respectively:

(5) $$\;\Pi =\frac{W_{t}}{\delta_{max}^{3}\cdot \psi_{\sigma }\cdot \sigma_{R}\langle 0.0115\rangle }=-0.20821\xi^{3}+2.6502\xi^{2}-3.7040\xi +2.7725\;$$

(6) $$\;\Omega \equiv \frac{S}{2\delta_{max}\bar{E}}=A\xi^{3}+B\xi^{2}+C\xi +D\;$$

where $\xi =\ln\left(\bar{E}/\left(\psi_{\sigma }\cdot \sigma_{R}\langle 0.0115\rangle \right)\right)$ with the plane strain modulus $\bar{E}=E/\left(1-\upsilon^{2}\right)$, Young’s modulus Em and Poisson’s ratio υ; and the representative stress is $\sigma_{R}$, with the representative strain of 0.0115 for a Berkovich indenter. The indentation work done is $W_{t}=\int_{0}^{\delta \max}Pd\delta$, determined by area integration from the beginning until the maximum penetration depth of $\delta_{\max}$ in the loading part, and the contact stiffness S is the initial unloading slope of the applied load–penetration depth curve. Both $W_{t}$ and S have been illustrated in Figure 2. It can be seen in Equation (5) that the maximum penetration depth $\delta_{\max}$ dominates the dimensionless variable Π, which is associated with the loading part. In Equation (6), for the unloading part, the dimensionless variable Ω is a function of $\vartheta =\psi_{n}\cdot n$, with the hardening exponent of n enriched by the rate factor of $\psi_{n}$, in a series of coefficients that are numerically obtained by extensive finite element simulations as follows: $A=-0.04783\vartheta^{2}+0.04667\vartheta -0.01906$, $B=0.6455\vartheta^{2}-0.6325\vartheta +0.2239$, $C=-2.298\vartheta^{2}+2.025\vartheta -0.4512$, and $D=2.050\vartheta^{2}-1.502\vartheta +2.109$.

**Figure 7:**
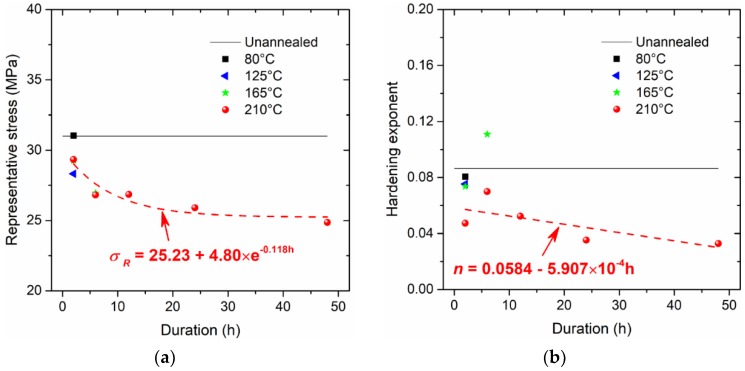
Determined properties from nanoindentation responses: (**a**) contact stiffness during the unloading stage, and (**b**) work done during the loading stage.

The value of indentation work done ($W_{t}$) and contact stiffness (S) can be determined, as shown in Figure 7, from the applied load–penetration depth curve in Figure 3, as directly recorded from the nanoindentation instrument. Unlike the random distributions for the other thermal treatments, the indentation work done on the samples annealed by the temperature of 210 °C follows a linear relationship with the duration, while the contact stiffness approaches a stable value of about 0.662 with the increasing annealing duration, at the temperature of 210 °C. The unknown variable $\sigma_{R}\langle 0.0115\rangle$ and n can be conveniently obtained by finding out the intersection of the two curves from the left and right sides of Equations (5) and (6), respectively. Then, the hardening rate R can be determined by substituting the parameters in a power-law constitutive equation for the representative strain $\varepsilon_{R}=0.0115$. It should be noted that a reduction of 30% is made for the value of contact stiffness, as the slope in the initial unloading part is difficult to be quantified, and the automatically recorded values are usually found to be artificially high. This minor assumption does not invalidate the physical basis of the adopted methodology, but ensures the existence of solutions to Equation (6) when solving the hardening exponent n based on Equation (6). 

**Figure 8:**
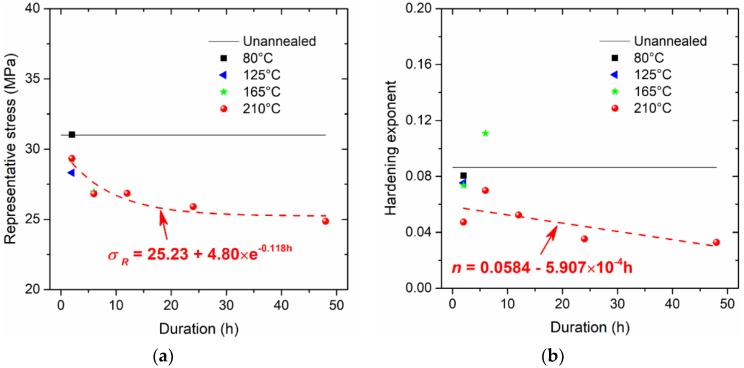

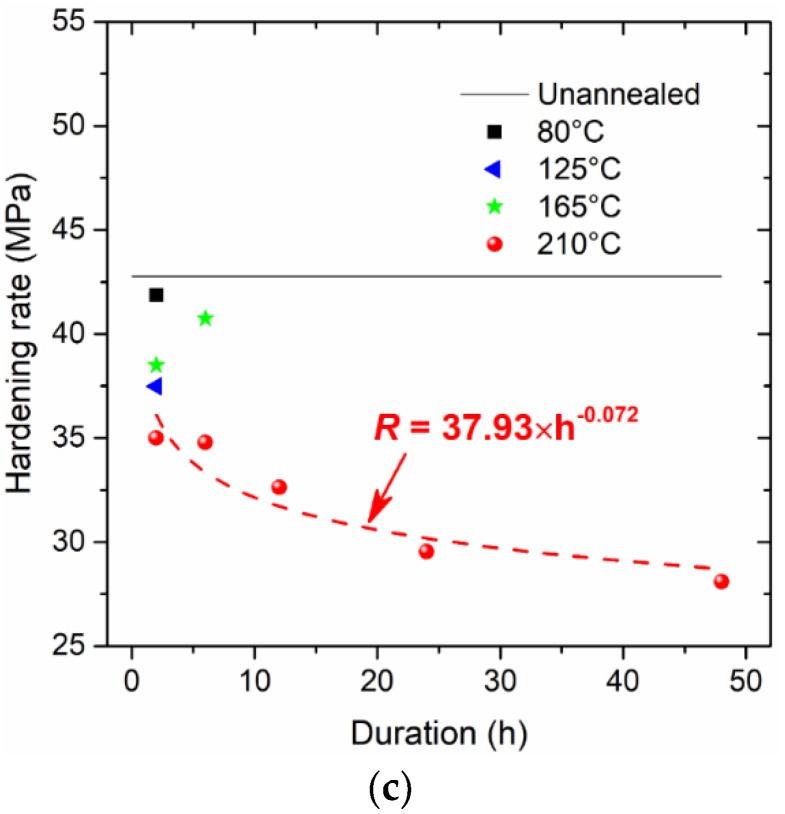
Critical parameters of constitutive model: (**a**) representative stress, (**b**) hardening exponent, and (**c**) hardening rate.

The determined values for the representative stress $\sigma_{R}$, the hardening exponent n, and the hardening rate R are provided in Figure 8. Apparently, with increasing duration at the annealing temperature of 210 °C, the representative stress is asymptotically approaching a stabilized value of about 25.23 MPa; the hardening exponent n approximately linearly decreases, and the hardening rate R follows a power-law equation as it decreases. The parameters in Figure 8 are well-described by some fitting formulae, which are therefore inferred to be physically meaningful with regards to dominating the constitutive behaviour.

**Figure 9:**
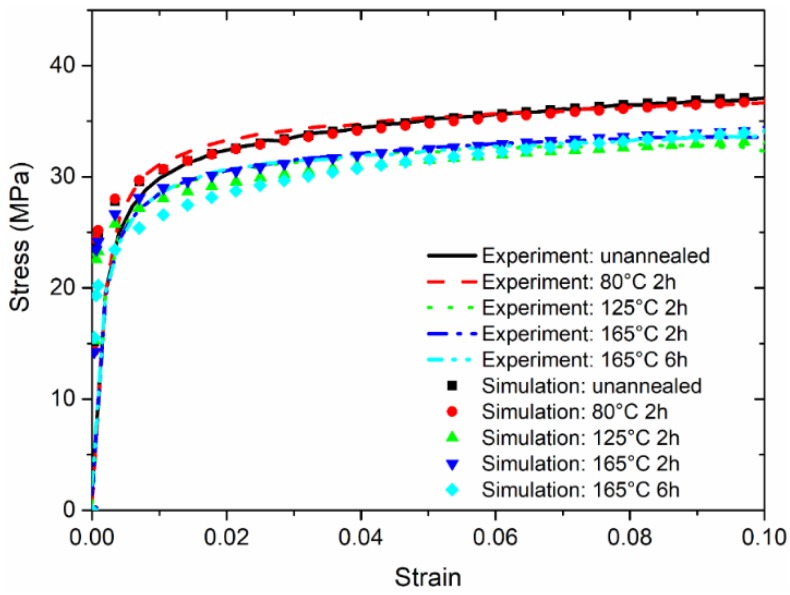
Comparison of stress–strain responses for the annealing temperatures below 210 °C.

**Figure 10:**
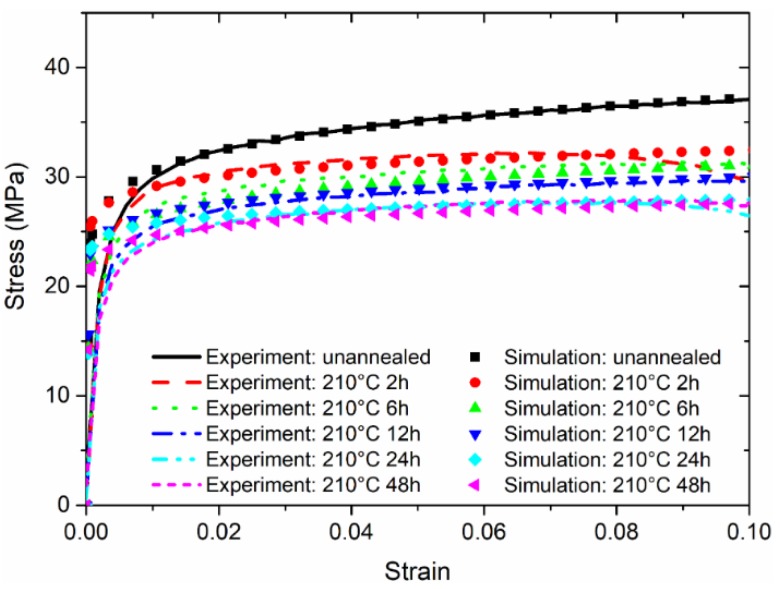
Comparison of stress–strain responses for the annealing temperatures at 210 °C.

**Figure 11:**
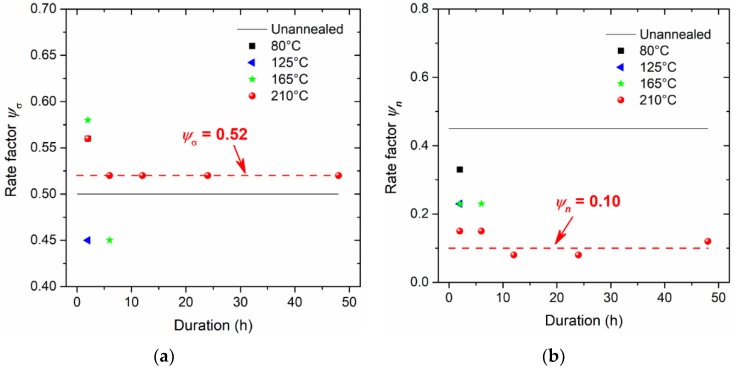
Rate factors for (**a**) representative stress and (**b**) the hardening exponent.

Figure 9 and Figure 10 show that good agreement can be achieved based on comparisons of stress–strain responses measured from tensile experiments and estimated from the applied load–indentation depth responses of nanoindentation experiments. Similar to the published works by Fu et al. [17], there is a certain discrepancy between the predicted and measured curves, especially for the elasto-plastic transition stage. This is very difficult to extract accurately in such a short regime, as explained by Patel and Kalidindi [36]. Nevertheless, in order to best reproduce the stress–strain relationship obtained from the tensile experiment, using the dog-bone type specimens at the strain rate of 5 × 10^−4^ s^−1^, the rate factors $\psi_{\sigma }$ and $\psi_{n}$ are determined in Figure 11 to enrich the parameters $\sigma_{R}$ and n in the nanoindentation experiments at the strain rate of 0.05 s^−1^. The general trend of the rate factor $\psi_{\sigma }$ is found to be stabilized at the value of 0.52, and the rate factor $\psi_{n}$ is about 0.10 if the duration is sufficient at the annealing temperature of 210 °C. It is apparent that for both tensile and nanoindentation experiments, the thermal treatments—especially at a sufficiently high temperature for the material sample—are important for stabilizing the mechanical behavior, and align the material property for both types of specimens. Thus, the proposed approach is believed to be reliable for estimating the stress–strain relationships from the nanoindentation responses. 

## 5. Conclusions

In this study, the constitutive behaviour from tensile and nanoindentation experiments was analytically correlated for SAC305 solder samples annealed by various temperatures and durations. Conclusions can be drawn as follows:

- A high annealing temperature close to the melting temperature, with a sufficient duration, benefits the alleviation of residual stress and the stabilization of microstructure, with fewer micro-defects. The constitutive behaviour of SAC305 solder annealed at 210 °C can be used for parameter calibrations.
- Rate factors $\psi_{\sigma }$ and $\psi_{n}$ are proposed and determined to be 0.52 and 0.10, to respectively multiply the representative stress and stress exponent for characterizing the integrated work done and the contact stiffness for the loading and unloading stages of nanoindentation responses.
- The proposed analytical methodology and rate factors can be applicable to other metals and alloys, provided that the material sample of interest is without significant residual stress.

Further studies will be considered to evaluate the generalized rate factors based on the dimensionless approach, so that the stress–strain relationships at a practical range of strain rates can be estimated by performing nanoindentations at a strain rate.
